# Spider Mites Singly Infected With Either *Wolbachia* or *Spiroplasma* Have Reduced Thermal Tolerance

**DOI:** 10.3389/fmicb.2021.706321

**Published:** 2021-07-07

**Authors:** Yu-Xi Zhu, Zhang-Rong Song, Yi-Yin Zhang, Ary A. Hoffmann, Xiao-Yue Hong

**Affiliations:** ^1^Department of Entomology, Nanjing Agricultural University, Nanjing, China; ^2^Institute of Applied Entomology, School of Horticulture and Plant Protection, Yangzhou University, Yangzhou, China; ^3^School of BioSciences, Bio21 Institute, The University of Melbourne, Melbourne, VIC, Australia

**Keywords:** *Wolbachia*, *Spiroplasma*, *Tetranychus truncatus*, thermal tolerance, thermal preference

## Abstract

Heritable symbionts play an essential role in many aspects of host ecology in a temperature-dependent manner. However, how temperature impacts the host and their interaction with endosymbionts remains largely unknown. Here, we investigated the impact of moderate (20°C) and high (30 and 35°C) temperatures on symbioses between the spider mite *Tetranychus truncatus* and two maternally inherited endosymbionts (*Wolbachia* and *Spiroplasma*). We found that the thermal tolerance of mites (as measured by survival after heat exposure) was lower for mites that were singly infected with either *Wolbachia* or *Spiroplasma* than it was for co-infected or uninfected mites. Although a relatively high temperature (30°C) is thought to promote bacterial replication, rearing at high temperature (35°C) resulted in losses of *Wolbachia* and particularly *Spiroplasma*. Exposing the mites to 20°C reduced the density and transmission of *Spiroplasma* but not *Wolbachia*. The four spider mite strains tested differed in the numbers of heat shock genes (*Hsps*) induced under moderate or high temperature exposure. In thermal preference (Tp) assays, the two *Wolbachia*-infected spider mite strains preferred a lower temperature than strains without *Wolbachia*. Our results show that endosymbiont-mediated spider mite responses to temperature stress are complex, involving a combination of changing endosymbiont infection patterns, altered thermoregulatory behavior, and transcription responses.

## Introduction

Temperature has a substantial impact on a wide range of ecologically important traits in many ectothermic organisms (Colinet et al., 2015; Sgro et al., 2016; Ma et al., 2021) and their symbiotic microbiota (Kiers et al., 2010). Heritable bacterial symbionts, which are pervasive in many arthropods, affect the host’s response to abiotic stressors, including temperature (Lemoine et al., 2020). Bacterial endosymbionts can both mediate and constrain host adaptation to temperature extremes (Corbin et al., 2017). For example, the obligate bacterial endosymbiont, *Buchnera*, limits the thermal tolerance of its aphid hosts and thus represents an “Achilles’ heel” of their thermal response (Dunbar et al., 2007; Zhang et al., 2019), while the facultative endosymbiont *Serratia symbiotica* increases aphid survival or reproduction under heat exposure likely through releasing metabolites (Montllor et al., 2002; Burke et al., 2010). Some strains of *Wolbachia* can increase *Drosophila melanogaster*’s thermotolerance by accelerating dopamine metabolism (Gruntenko et al., 2017), while *Hamiltonella* infections in whitefly also confer a fitness advantage under heat that may involve induction of the expression of host-produced stress genes (Brumin et al., 2011). Thermal effects mediated by symbionts may therefore affect host distribution as well as responses to ongoing changing climate.

Conversely, the persistence and stability of the bacterial symbionts themselves can be affected by temperature stress (Wernegreen, 2012), which can impact facultative endosymbiont dynamics through influencing their density, transmission, and phenotypic effects (Renoz et al., 2019). For instance, heat stress from exposure to high temperatures (>30°C) reduces *Wolbachia* density in various species, including *Aedes aegypti* (Ross et al., 2020), *D. simulans* (Hoffmann et al., 1986), and *Nasonia vitripennis* (Bordenstein and Bordenstein, 2011), while in aphids it affects densities of the nutritional obligate symbiont *Buchnera* (Zhang et al., 2019). Reduced *Wolbachia* density results in weaker cytoplasmic incompatibility (CI) which assists endosymbionts like *Wolbachia* to spread in populations, and reduced endosymbiont density can also lead to maternal transmission failure (Ross et al., 2017). Other host species such as *Acyrthosiphon pisum*, that have a complex mixture of symbionts, can show a recovery of their obligate symbionts after heat stress that depends on their facultative symbionts (Heyworth et al., 2020). Thus, impacts of thermal conditions on symbionts may vary depending on the nature of the stress, host species, and symbiont community.

The spider mite, *Tetranychus truncatus*, is a dominant mite species in China (Jin et al., 2018) and is frequently associated with multiple endosymbionts including *Wolbachia* and *Spiroplasma* (Zhu et al., 2018). Both these symbionts are maternally inherited and can co-infect the same *T. truncatus* host. *Wolbachia* induces incomplete CI in spider mites (Yang et al., 2020). A comprehensive survey of endosymbionts in the natural population of *T. truncatus* has shown that the incidence of endosymbionts is associated with environmental factors. In particular, *Wolbachia* infection rates increase at localities with higher annual mean temperatures (Zhu et al., 2018). This is in contrary to high temperatures negatively impacting *Wolbachia* transmission and CI strength in the related mite T. *urticae* (van Opijnen and Breeuwer, 1999), which may be correlated with the lytic activity of phage WO (Lu et al., 2012). *Wolbachia*-induced CI phenotype and transmission are therefore temperature dependent in spider mites; however, the impact of temperature on endosymbiont interactions is not clear in *T. truncatus*.

In the present study, we investigated the impact of high and low temperatures on the survival of females from *T. truncatus* strains that were either uninfected or singly and doubly infected with *Wolbachia* and *Spiroplasma* strains. We also tested the effect of temperature stress on endosymbiont density and vertical transmission efficiency, and we explored the transcriptome to investigate possible mechanisms associated with any endosymbiont-mediated temperature responses. Finally, we tested whether infection with *Wolbachia* and *Spiroplasma* affected host thermal preference (Tp). We hypothesized that spider mites infected with different symbiont strains would display differences in thermal tolerance and Tp; in turn, symbioses of *Wolbachia* and *Spiroplasma* with *T. truncatus* would be affected by temperature stress.

## Materials and Methods

### Spider Mite Strains and Rearing

Four spider mite strains with a similar genetic background were established (Yang et al., 2020; Zhu et al., 2020): strains co-infected with *Wolbachia* and *Spiroplasma* (designated as *w* + *s* +), *Wolbachia* only (*w* +), *Spiroplasma* only (*s* +), or no symbionts (*w-s-*). Symbiont-infected mite strains (*w* + *s* + , *w* +, and *s* +) were collected from Shenyang, Liaoning Province, China. A *w-s-* strain was obtained by tetracycline treatment of the *s* + strains for three generations. To eliminate potential effects of the tetracycline, we cultivated the *w-s-* strain without tetracycline treatment for 15 generations. To minimize genetic background differences between four spider mite strains, we mated infected female adults with the uninfected male adults and backcrossed female offspring for at least seven consecutive generations.

Spider mite strains were reared on leaves of the common bean (*Phaseolus vulgaris* L.) placed on a water-saturated sponge mat in a Petri dish at 25 ± 1°C and 60% relative humidity and under 16 h light: 8 h dark conditions. Prior to the experiment, the infection status was checked as by PCR (Yang et al., 2020). A schematic of the study design is given in Supplementary Figure 1.

### Survival at Constant Temperatures

To determine survival of the four strains following different temperature treatment, we exposed 20 female adults (2-day) to either 20, 25, 30, or 35°C in an incubator and then monitored spider mite survival daily for a week using a stereo microscope. Six biological replicates for each treatment were tested.

### Crossing Experiments

*Wolbachia*-induced incomplete CI was determined through crosses as previously described (Yang et al., 2020). To test the impact of different temperatures on the strength of CI, females without *Wolbachia* (♀: *s* + or *w-s-*) were crossed with males infected with *Wolbachia* (♂: *w* + *s* + or *w* +), producing a total of four crosses. Single females in the teleiochrysalis stage and a 1-day-old adult virgin male were placed on the same leaf disk, which was subjected to one of four temperature treatments: 20, 25, 30, or 35°C. Each cross and treatment combination was replicated 18–99 times using different leaf disks. Females were allowed to lay eggs for 5 days while maintaining treatment temperatures, and then removed. Eggs on leaf disks were continued to be kept under their respective treatment temperatures and were checked daily to calculate hatchability.

### Transmission Efficiency of *Wolbachia* and *Spiroplasma*

To examine the vertical transmission of *Wolbachia* and *Spiroplasma*, spider mites from each strain were placed on bean leaf discs (diameter ca. 3 cm) and were reared for four generations under 20, 25, 30, or 35°C. Twenty female adults (2 days old) were randomly selected from each treatment and generational combination (F1 to F4) of these cultures and used in PCR analyses. Six biological replicates were set up per strain. DNA was extracted from a single mite using a DNeasy blood and tissue kit (Qiagen, Hilden, Germany) according to the manufacturer’s protocols. *Wolbachia* and *Spiroplasma* infections were screened by PCR amplification using the specific primers listed in Supplementary Table 1. PCRs were carried out using a Veriti thermocycler (ABI Biosystems, United States) in a 25 μl volume containing 12.5 μl 2 × rapid Taq master mix (Vazyme Biotech, China), 0.5 μl primers (20 μM each), and 1 μl of DNA extract. PCR cycling parameters were 95°C for 3 min, followed by 35 cycles of 95°C for 15 s, the annealing temperature for 45 s, and 72°C for 15 s, and then 72°C for 5 min at the end. PCRs included a positive and negative control and were run on a 1% agarose gel with ethidium bromide to visualize the product.

### Densities of *Wolbachia* and *Spiroplasma*

To estimate the dynamics of *Wolbachia* and *Spiroplasma* following different temperature exposures, the ratio of the single-copy genes *16S rRNA* (*Spiroplasma*) and *wsp* (*Wolbachia*) to the *rps-18* (spider mite host) reference gene was determined by real-time qPCR (Yang et al., 2020). Spider mites subjected to the four different temperatures were sampled at 6, 12, and 24 h, as well as on the third and fifth days. For each time point, nine biological replicates were tested per treatment. For each of the biological replicates, we performed three technical replicates. Densities of *Wolbachia* and *Spiroplasma* were estimated by qPCR with the ABI QuantStudio 6 Flex (Applied Biosystems, CA, United States). The 20-μl Q-PCR reaction mixture consisted of 10 μl 2 × SYBRP remix Ex Taq (Vazyme, China), 0.4 μl 10 mmol/L of each primer, 0.4 μl 50 × ROX Reference Dye, 2 μl DNA template, and 6.8 μl H_2_O in single wells of a 96-well plate (PE Applied Biosystems, CA, United States). The Q-PCR cycling conditions included one cycle (5 min at 95°C) followed by 40 cycles (10 s at 95°C and 34 s at 60°C), and finally one cycle to produce melting curves (15 s at 95°C, 1 min at 60°C, and 15 s at 95°C). The primers are listed in Supplementary Table 1. Standard curves were plotted using a 10-fold dilution series of the DNA samples prepared from plasmid DNA. The plasmid DNA was obtained with the pEASY-T3 vector (TransGen Biotech, Beijing, China). The quality and concentration of all purified standard DNA were measured on a Nanodrop 2000 (Thermo Scientific, MA, United States).

### Transcriptome Processing and Annotation

To explore transcriptional responses to short-term high or low temperature exposure in the four spider mite strains, we sampled 2-day-old female adults with exposure to either 20, 25, 30, or 35°C for 6 h. About 100 adult female spider mites (2-day-old) were collected for each replicate and then frozen in liquid nitrogen prior to RNA extraction. Four biological replicates for each treatment were tested.

Total RNA was extracted from each sample using Trizol protocol according to manufacturer’s instructions (Invitrogen, CA, United States). RNA quality was qualified and quantified using a Nano Drop and Agilent 2100 bioanalyzer (Thermo Fisher Scientific, MA, United States). Construction of cDNA libraries and subsequent sequencing using the BGIseq500 platform were conducted at BGI-Shenzhen, China. Raw data were filtered using SOAPnuke v1.5.2 by removing reads containing adapters, poly-N, and low-quality reads to obtain clean data. Different gene expression was analyzed using the DESEQ2 package (Love et al., 2014) with *Q* value ≤ 0.05. Kyoto Encyclopedia of Genes and Genomes (KEGG) enrichment analysis and Gene Ontology (GO) were based on the KEGG pathway database^1^ and the GO database^2^.

To confirm the results of the RNA-seq analysis, the expression levels of randomly selected genes were determined by RT-qPCR. The RT-qPCR reactions were performed on an ABI QuantStudio 6 Flex Real-Time PCR System with SYBR Premix Ex Taq (Takara Bio, Kyoto, Japan). The amplification reactions were performed in a 20 μL final volume containing 10 μL of TaKaRa buffer, 7.2 μL of ddH_2_O_2_, 0.4 μL of DyeII (TaKaRa), 0.2 μL of forward primer (5 mM) and reverse primer (5 mM), and 2 μL of cDNA first-strand template. Thermal cycling conditions were as follows: 95°C for 10 min, then 40 cycles of 5 s at 95°C, and 34 s at 60°C. There were three technical replicates for each sample. Primer sequences were designed using Primer Premier 6.0, and are listed in Supplementary Table 1. Expression levels for each gene were calculated by the 2^–ΔΔ*CT*^ method (Livak and Schmittgen, 2001).

### Thermal Preference Assays

Temperature preference assays were performed using a custom-built thermal gradient apparatus consisting of a 500-mm-long aluminum bar along which a temperature gradient (10–40°C) created with water baths at the ends. Six grooves were etched along the aluminum bar which allowed mites to move freely up or down the gradient without interacting with each other (Supplementary Figure 2). All assays were conducted in a room with a constant temperature of 25°C and constant 40% humidity. For each data point, six individuals of the same strain were, respectively, transferred to the center point of each groove in the apparatus (25°C) and allowed to move freely along its groove for 30 min. The temperatures at the positions of rest for the six spider mites after 30 min, as measured with K-type thermocouples, were used to calculate a mean preferred temperature. In total, more than 282 individuals from each spider mite strain were used to test host Tp.

### Statistical Analysis

All statistical analyses were carried out in R ver 3.3.1 or GraphPad Prism 9.0 (GraphPad Software Inc., San Diego, CA, United States). Log-rank (Mantel–Cox) tests were used to compare the survival proportions of the spider mite strains under different temperatures. We also compared differences in survival rate between each treatment at the same time point using two-way ANOVAs with multiple comparisons. We used either ANOVAs or Mann–Whitney tests to compare endosymbionts densities, egg hatch proportions, and frequencies of endosymbiont infection, depending on whether data were normally distributed or deviated from normality based on Kolmogorov–Smirnov tests. We conducted a non-parametric Kruskal–Wallis test to compare the Tp values. Differences were considered significant at *p* < 0.05 for all analyses.

## Results

### Singly Infected *Wolbachia* or *Spiroplasma* Strains Have Lower Fitness Than a Co-infected Strain at Higher Temperatures

**FIGURE 1:**
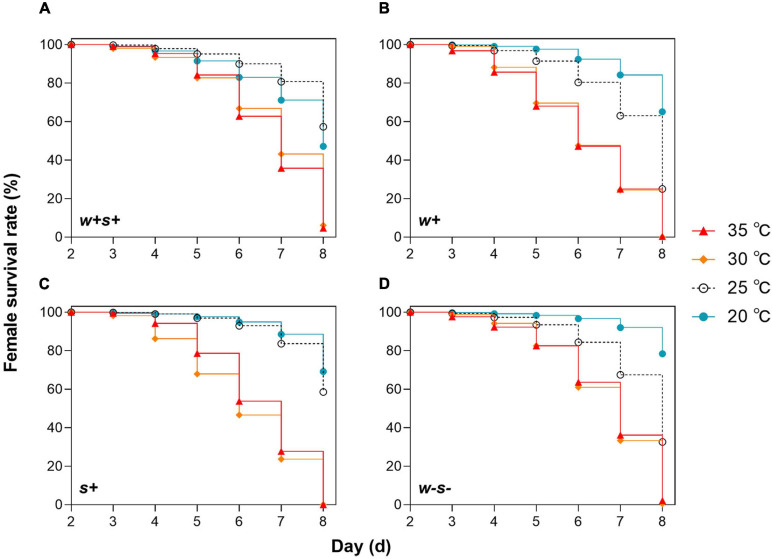
Survival rates of four spider mite strains over 8 days under different temperatures. **(A)** *Wolbachia* and *Spiroplasma* co-infected strains (*w*+*s*+), **(B)** singly *Wolbachia*-infected strains (*w*+), **(C)** singly *Spiroplasma*-infected (*s*+), and **(D)** uninfected stains (*w-s-*).

We first compared female survival for mites reared under either 20, 25, 30, or 35°C. The survival rate of four spider mite strains was significantly (*p* < 0.0001) affected by temperature (log-rank test, *w* + *s* + : χ^2^ = 311.2, *df* = 3; *w* + : χ^2^ = 660.8, *df* = 3; *s* + : χ^2^ = 885.0, *df* = 3; *w-s-*: χ^2^ = 522.5, *df* = 3; Figure 1). After 2–4 days, the female survival rates of co-infected strains were not significantly different at 25, 30, and 35°C, but after 4 days, the female survival rate of the other three strains was significantly lower at the higher temperatures (30 and 35°C) than at 25°C (Figure 1). This suggests that co-infection with *Wolbachia* and *Spiroplasma* delayed high temperature effects on the host. Moreover, when the four mite strains were reared at 30 and 35°C, the female survival rate after 5 days was sharply lower in the singly infected strains than in the co-infected and uninfected strains (Figure 1). These results indicate that spider mites singly infected with *Wolbachia* or *Spiroplasma* are sensitive to high temperatures. In contrast, female survival rate did not differ significantly between 20 and 25°C for either co-infected or *Spiroplasma*-infected strains at any time point. However, survival did sharply decrease at 25°C in *Wolbachia*-infected and uninfected strains after 5 days (Figure 1). Spider mites infected with *Spiroplasma* may therefore have a higher fitness at the intermediate temperature.

### Higher and Lower Temperatures Modify the CI Strength of *Wolbachia*

**FIGURE 2:**
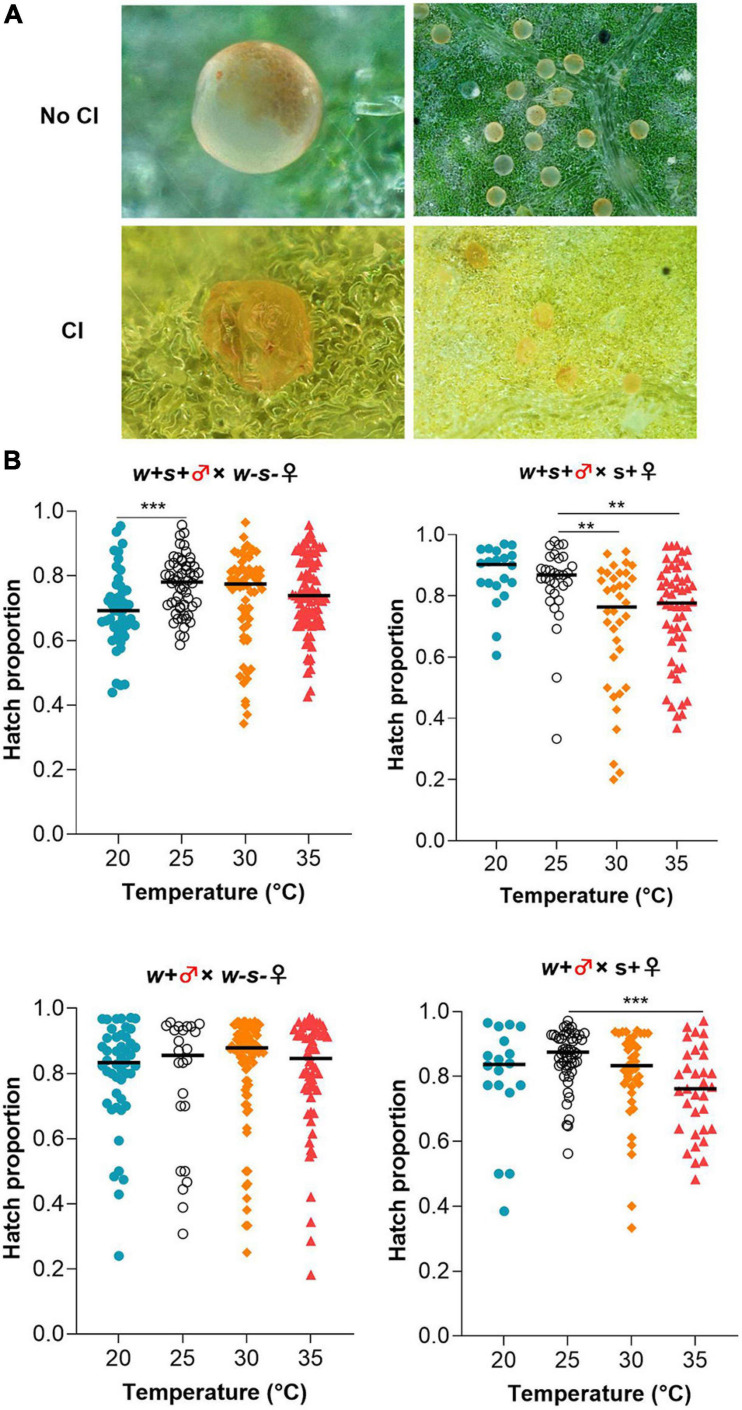
Effect of low (20°C) and high (30 or 35°C) temperatures on *Wolbachia-*induced CI. **(A)** Visual differences in normal eggs (upper panels) and stunted eggs from CI (lower panels). **(B)** Egg hatch rates in four crosses between different spider mite strains at either kept under different temperatures. Horizontal bars indicate the medians based on *n* = 18–99 per treatment. Mann–Whitney test, **p < 0.01, ***p < 0.001.

Consistent with previous results (Yang et al., 2020), we found that *Wolbachia* induced incomplete CI, resulting in 20–30% offspring mortality when mites were reared at 25°C. Embryos showed aborted development when CI occurred (Figure 2A). Exposure to a high temperature (35°C) resulted in a significant decrease in the mean egg hatch rate in the cross between *Spiroplasma*-infected females and co-infected or *Wolbachia*-infected mites compared to those same crosses at 25°C (Mann–Whitney test, *w* + *s* + ♂ × *s* + ♀: 35°C vs. 25°C, *p* = 0.0025; *w* + ♂ × *s* + ♀: 35 vs. 25°C, *p* = 0.0002) (Figure 2B). The hatch proportion significantly declined at the lower temperature in the cross between co-infected males and uninfected females at 20°C compared to those at 25°C, from 77.78% at 25°C to 69.39% at 20°C (Mann–Whitney test: *p* = 0.0004). For the crosses between uninfected females and *Wolbachia*-infected males, there was no significant difference in the hatch proportion of offspring exposed to either 20, 25, 30, or 35°C (Figure 2B).

### Higher Temperatures Affect Endosymbiont Titer in Female Hosts

**FIGURE 3:**
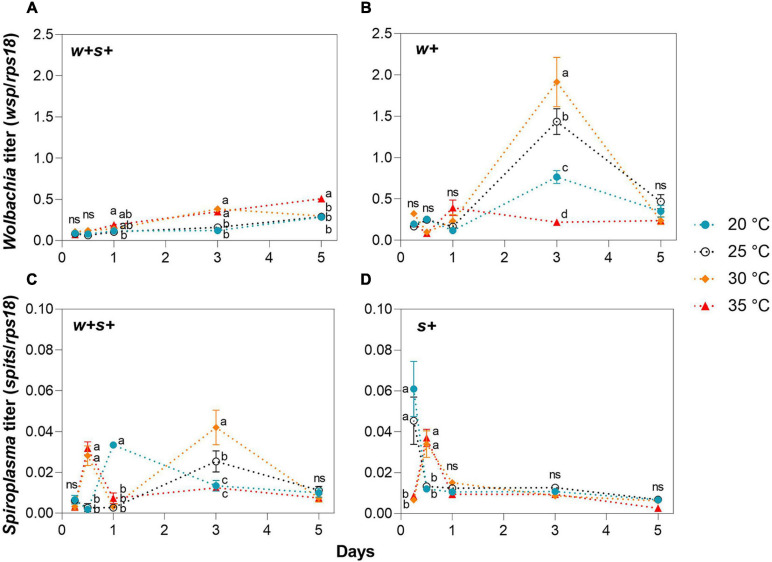
*Wolbachia* and *Spiroplasma* densities in female adult spider mites maintained under low (20°C) and high (30 or 35°C) temperatures for up to 5 days. *Wolbachia* density in co-infected strains **(A)** and *Wolbachia*-infected strains **(B)**. *Spiroplasma* density in co-infected strains **(C)** and *Spiroplasma*-infected strains **(D)**. Data are shown as the mean ± SEM. Different letters indicate significant differences between each treatment at the same time point (*p* < 0.05; ns, no significant).

We measured the effect of temperature stress on *Wolbachia* and *Spiroplasma* density in female hosts. *Wolbachia* titers in co-infected strains exposed to 35°C were significantly higher than in these strains at 25°C at days 1, 3, and 5, but no significant difference between strains was found at 20 and 25°C at all time points (Figure 3A). In contrast to co-infected strains, *Wolbachia* titers in *Wolbachia* singly infected strains at either 20 and 35°C were significantly lower than those at 30 or 25°C at day 3 (Figure 3B). *Spiroplasma* densities in both co-infected and *Spiroplasma*-infected strains exposed to either 30 or 35°C were significantly higher than those strains at either 20 or 25°C at day 0.5 (Figures 3C,D). *Spiroplasma* densities in co-infected strains fluctuated at later time points under high and low temperatures, while there were no significant differences in *Spiroplasma* densities in singly infected strains among temperature treatments at days 1, 3, and 5 (Figures 3C,D). The average titer and the shifts in titer with age varied across spider mite strains.

### High Temperatures Reduce Infection Frequency of Both *Wolbachia* and *Spiroplasma*

**FIGURE 4:**
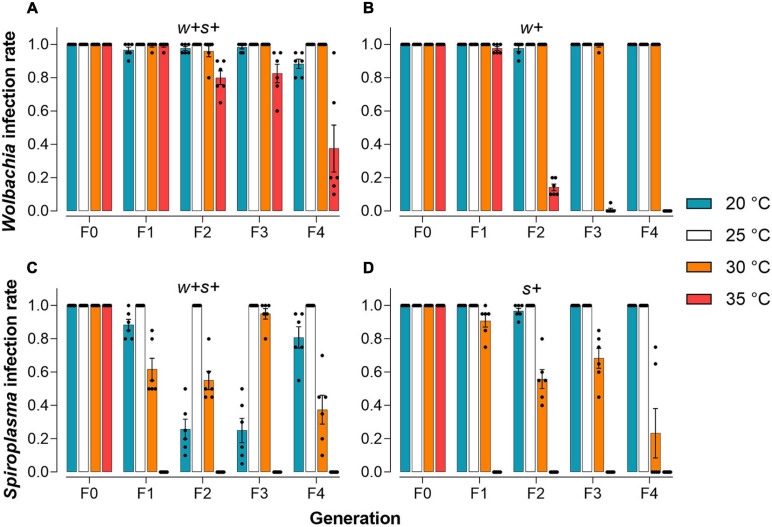
Infection frequencies of two endosymbionts in female adult spider mites reared for four generations under different temperature conditions. Infection rate of *Wolbachia* in co-infected strains **(A)** and *Wolbachia*-infected strains **(B)**. Infection rate of *Spiroplasma* in co-infected strains **(C)** and *Spiroplasma*-infected strains **(D)**.

We tested the *Wolbachia* or *Spiroplasma* infection frequency in co-infected and singly infected strains over four generations when mites were held at 20, 25, 30, or 35°C. At 25°C, the normal rearing temperature for host spider mites, both *Wolbachia* and *Spiroplasma* were stably maintained with 100% prevalence in either co-infected or singly infected strains through successive host generations (Figures 4A–D). However, *Spiroplasma* was lost in both co-infected and singly infected strains when mites were reared at 35°C for one generation (Figures 4C,D). The prevalence of *Wolbachia* in singly infected strains sharply decreased from 97.5% in the F1s to 14.2% in the F2s and the infection was nearly lost in later generations, but in co-infected mites, the reduction at 35°C was less (Figures 4A,B). At 30°C, *Wolbachia* maintained nearly 100% prevalence in both co-infected and singly infected strains. In contrast, the prevalence of *Spiroplasma* in singly infected strains decreased from 90.8% in the F1s to 55.8% in the F2s, while the prevalence of *Spiroplasma* in co-infested strains fluctuated across the four generations (Figures 4C,D). These results suggest that high temperature reduces vertical transmission efficiency of *Wolbachia* or *Spiroplasma* in all spider mite strains with varying degrees, and *Spiroplasma* is more sensitive to high temperatures than *Wolbachia*. Exposure to a cooler temperature (20°C) did not affect the infection frequencies of *Wolbachia* and *Spiroplasma* in singly infected strains. However, the *Spiroplasma* infection rate at this temperature in co-infected strains rapidly decreased from 88.3% in the F1s to a low level of 25% in the F3s, before increasing in later generations to 80.8%. The *Wolbachia* infection also varied somewhat in the co-infected strain across generations when mites were held at 20°C (Figure 4A).

### Gene Expression Differentiation Among Four Spider Mite Strains Under High and Low Temperatures

**FIGURE 5:**
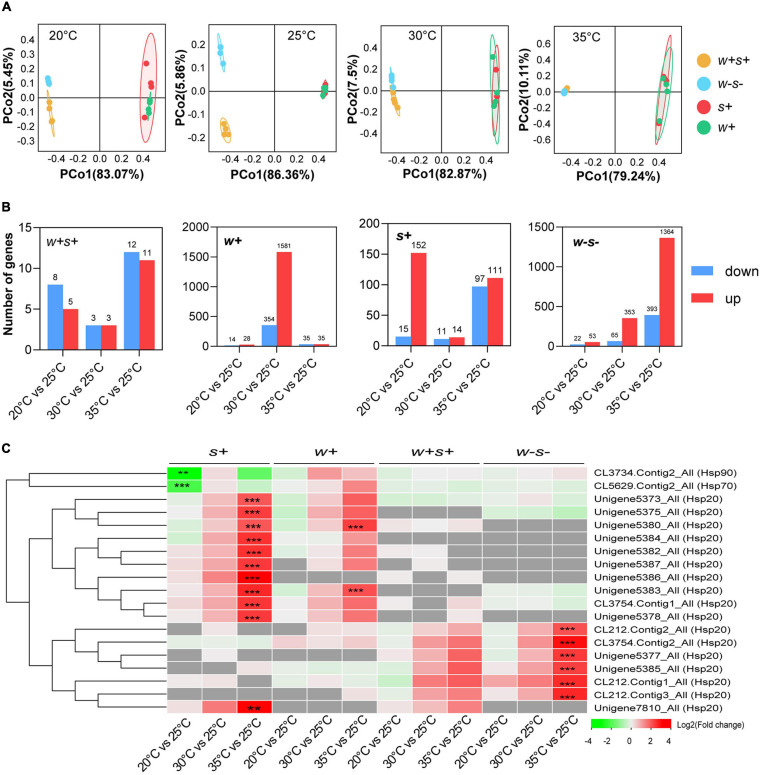
Transcription response of four female adult spider mite strains following 6 h of different temperature exposures. **(A)** Principal components analysis (PCoA) of gene expression patterns of four spider mite strains under 20, 25, 30, and 35°C. **(B)** Number of differentially expressed genes (DEGs) with fold change > 2 and an FDR-adjusted *p*-value of < 0.05 for four spider mite strains under different temperatures. **(C)** Significantly differently expressed *Hsp* genes were analyzed by comparing each of 20, 30, and 35°C vs. the control (25°C) based on a threshold of > 2-fold change and an FDR-adjusted *p*-value of < 0.05. The gray squares in the heat map indicate that gene expression was absent. Asterisks indicate statistically significant differences (*p < 0.05; **p < 0.01; and ***p < 0.001).

Comparison of transcriptional responses to high and low temperatures was based on a total of 2,685.47 Mb clean reads from 63 libraries derived from four spider mite strains (Supplementary Table 1). Unigenes were annotated to the KEGG and GO databases (Supplementary Figure 3). The PCoA analysis explained 87.88% of the variance in gene expression of the four spider mite strains under different temperatures (Figure 5A). PCoA demonstrated that the expression patterns of the singly infected *Wolbachia* strain and singly infected *Spiroplasma* strain were closely related at all temperatures (Figure 5A).

Variation in gene expression between temperatures was analyzed through comparisons of 35 vs. 25°C, 30 vs. 25°C, and 20 vs. 25°C. The doubly infected strain had the fewest differentially expressed genes compared with the other three mite strains under both high and low temperature exposure (Figure 5B—note scale differences). For the low temperature (20°C) comparison, *Spiroplasma*-infected strains showed the most differently expressed genes (DEGs). For 35°C comparison, the number of DEGs in un-infected strains was higher than that in the other three spider mite strains when compared to the control treatments.

Among the DEGs, expression of the heat shock protein genes, including *Hsp20*, *Hsp70*, and *Hsp90*, varied in particular between the four spider mite strains. Within the *Hsp20* superfamily, a total of 11, two, and six orthologous genes were strikingly up-regulated in *Spiroplasma*-infected, *Wolbachia*-infected, and uninfected spider mite strains when spider mites were reared at 35 vs. 25°C. In contrast, two *Hsp* genes, *Hsp70* and *Hsp90*, were significantly down-regulated in *Spiroplasma*-infected strains under low temperature exposure (Figure 5C). We selected eight genes for RT-qPCR validation of differential expression in all samples. The results showed a concordant direction of change between the qPCR and transcriptomic analyses (Supplementary Figure 4).

### Effects of Endosymbionts on Thermal Preference in Spider Mite

**FIGURE 6:**
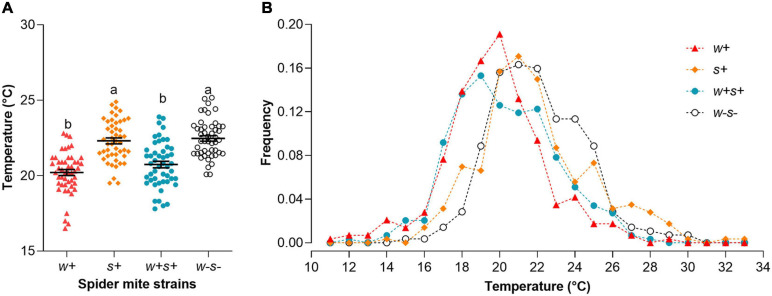
Thermal preference (Tp) of the four spider mite strains. **(A)** Average Tp of the four spider mite strains. Each dot on the graph indicates the mean of six individuals in each replicate. Long bar indicates the mean of all dots in the strain, and error bars indicate SE. Different letters indicate significant differences between four spider mite strains (*p* < 0.05). **(B)** Relative proportions of the four spider mite strains observed at a given temperature.

We used a custom-built thermal gradient to test whether *Wolbachia* and *Spiroplasma* affected temperature preference. The four spider mite strains exhibited a large variance in Tp, falling between 11.80 and 33.40°C (Figure 6A). There was some overlap in Tp distributions between the four strains (Figure 6B). However, the preferred average temperature of *Wolbachia* and *Spiroplasma* co-infected strains (Tp = 20.74 ± 0.22°C) and *Wolbachia*-infected strains (Tp = 20.21 ± 0.20°C) were significantly lower than either *Spiroplasma*-infected (Tp = 22.31 ± 0.20°C) or uninfected strains (Tp = 22.47 ± 0.18°C) (Kruskal–Wallis statistic: 67.67, *p* < 0.0001; Figure 6A), representing a difference of around 2°C. The results showed that spider mites infected with *Wolbachia* preferred cooler temperatures than uninfected spider mites.

## Discussion

In this study, we found that thermal tolerance was lower for mites that were singly infected with either *Wolbachia* or *Spiroplasma* than for co-infected and uninfected mites (Supplementary Table 3). It is likely that spider mites singly infected with either *Wolbachia* or *Spiroplasma* have reduced thermal tolerance, but co-infection by the two endosymbionts confers a level of protection for their hosts. Host fitness costs associated with high-temperature stress appear to be magnified when spider mites are singly infected with either *Wolbachia* or *Spiroplasma* (Corbin et al., 2017). In contrast, the co-infecting endosymbionts may additively or synergistically confer greater tolerance in their host (Vautrin and Vavre, 2009) which may improve performance under stressful environmental conditions (Renoz et al., 2019).

While underlying mechanisms remain unclear, researchers have previously documented that endosymbionts are vulnerable to both high- and low-temperature stress and that temperature-challenged insects with a disrupted symbiotic system often show elevated mortality and other defective phenotypes (Prado et al., 2010). Symbioses of *Wolbachia* and *Spiroplasma* with *T. truncatus*, like other heritable symbioses, appear vulnerable to temperature stress (Corbin et al., 2017). High temperatures can reduce endosymbiont density and even eliminate symbionts in various insects (Doremus et al., 2019). *Wolbachia* density can be reduced and lost within three generations when *T. urticae* is reared at 35°C (van Opijnen and Breeuwer, 1999). This also appears to be the case for *T. truncatus*, where *Wolbachia* is reduced over generations when the host is reared at 35°C but remains stable at 20 and 25°C. *Spiroplasma* is sensitive to both warmer and cooler conditions in co-infected spider mites when compared to the normal rearing temperature of 25°C. A similar phenomenon has been observed in *D. nebulosa*, where *Spiroplasma* was rapidly lost at 18°C and gradual loss occurred at 28°C (Anbutsu et al., 2008). Taken together, these results suggest that *Wolbachia* infections in mites differ from *Spiroplasma* in their response to low- and high-temperature stress, in that (i) *Spiroplasma* are more susceptible to high temperature than *Wolbachia* in either co-infected or singly infected strains, possibly due to *Spiroplasma* density being lower than *Wolbachia* density, and (ii) *Wolbachia* are more stable under high temperatures in doubly infected strains than in singly strains, whereas *Spiroplasma* seem more cold-sensitive in doubly infected strains than in singly infected strains. These patterns are consistent with the notion that temperature affects symbiont density and transmission in different ways, depending on the symbiont, host, and nature of the co-infection (Renoz et al., 2019).

Intriguingly, at both 30 and 20°C, the frequency of *Spiroplasma* in co-infected strains decreased at the second generation and then fluctuated in later generations. When one of the co-infected endosymbionts causes CI, this helps to maintain not only the CI-causing endosymbiont but also any co-infecting endosymbiont *via* a hitchhiking effect (Engelstadter et al., 2004). *Wolbachia* induced incomplete CI in co-infected *T. truncatus* strains. Thus, the *Spiroplasma* infection which exhibited an improvement under high or low temperature in co-infected strains may be hitchhiking with co-infecting *Wolbachia* that induce CI. This also partly helps to explain why co-infections with multiple endosymbionts are common in natural populations of *T. truncatus* (Zhu et al., 2018).

Temperature-induced changes to endosymbionts densities are likely to have cascading effects. For example, high temperature decreased *Wolbachia* and *Cardinium* density and thus decreased CI levels in *N. vitripennis* and Euphorbia *suzannae* (Bordenstein and Bordenstein, 2011; Doremus et al., 2019). In contrast, *Wolbachia* quickly replicate in *D. simulans* (Clancy and Hoffmann, 1998) and *Leptopilina heterotoma* wasps (Mouton et al., 2006) at warmer temperature, and yet CI strength decreases. Thus, complex interactions between symbiont densities and temperature influence CI strength variation, and this process may involve other factors such as host behavior, development, host genetic variation (Shropshire et al., 2020), and especially activity of the CI enzyme CidB, which also varies with temperature (Beckmann et al., 2019). Here, we found that high and low temperatures may impact *Wolbachia* density and *Wolbachia* CI strength; however, there was no direct association between CI strength and *Wolbachia* density (Yang et al., 2020). In *T. urticae*, high temperature also reduced *Wolbachia* densities, but CI strength may associate with an increase in phage WO lytic activity (Lu et al., 2012). This remains to be tested in *T. truncatus*.

In various heritable symbiotic systems, the thermal sensitivity of bacterial symbionts is likely to contribute to their ability to establish and persist in natural populations (Wernegreen, 2012). *Wolbachia* frequencies are high in spider mites across China and increase at localities with higher annual mean temperatures, while *Spiroplasma* is present at relatively low frequencies and patchily distributed (Zhu et al., 2018). The temperature conditions examined in this study are relevant to natural conditions experienced by mites in the field, although conditions there are variable, and it would be worth carrying out additional experiments under fluctuating temperatures (Ross et al., 2017).

We report for the first time that *Wolbachia* modifies Tp in spider mites, which might represent a behavioral accommodation to host–symbiont interactions (Truitt et al., 2019). Recently, *Wolbachia* strains in some *Drosophila* species were shown to prefer cooler temperatures (Arnold et al., 2019; Truitt et al., 2019; Hague et al., 2020), consistent with the current results. Differences in Tp between infected and uninfected flies could result from conflicting physiological requirements of *Wolbachia* and hosts (Hague et al., 2020), and this notion is worth further examination. We speculate that, as a consequence of trade-offs in thermal adaptation and balancing selection between the symbiont and the host, *Wolbachia*-mediated host thermoregulatory behavior may affect mite thermoregulation, endosymbiont spread, and the maintenance of facultative symbioses.

The main molecular mechanisms of symbiont-mediated effects on host tolerance may involve endosymbiont-induced changes in stress-response genes (Brumin et al., 2011). Among these genes, *Hsp* genes such as *Hsp*70, *Hsp*90, and small heat shock protein (*sHsp*) have been reported to play roles in high-temperature responses (Dahlgaard et al., 1998; Chen and Wagner, 2012). These proteins are molecular chaperones that promote correct refolding and prevent aggregation of denatured proteins in the face of a variety of stress factors, and thus may lead to host thermotolerance (King and MacRae, 2015). In the current study, the four spider mite strains differed in the expression changes of heat shock genes when exposed to different temperatures. The low number of DEGs in comparisons with the doubly infected strain suggests that co-infected strains are the least sensitive to different temperatures. In addition, *Spiroplasma* seems to interact with the relative expression of *Hsp70* and *Hsp90* at 20°C. It is not yet clear if the differences in host gene expression are a consequence of responses to temperature, temperature effects on the endosymbionts, or some combination of these factors. The few DEGs in the doubly infected strain may reflect a relatively higher tolerance of this strain to high temperatures, so that host *Hsps* were not triggered to the same extent. Alternatively, symbiont encoded genes may affect host tolerance and directly affect host gene expression. For example, mutants of the symbiont *Buchnera aphidicola* with lower expression of the heat-shock gene *ibpA* decrease heat tolerance of pea aphids (Zhang et al., 2019). Horizontal gene transfer (HGT) events between the bacteria and the host may also play a role and have recently been documented (Husnik et al., 2013; Husnik and McCutcheon, 2018). For instance, several genes horizontally transferred from diverse bacterial symbionts are known to be expressed in the host and can assist or enhance specific metabolic activities to promote host development (Manzano-Marín et al., 2020; Zhou et al., 2021), thus indirectly changing the thermal tolerance of the host. It is not clear whether HGT events between spider mites and endosymbionts produce similar effects.

## Conclusion

In summary, we found that *Wolbachia* and *Spiroplasma* in spider mites may affect the fitness of spider mites at different temperatures. We also show that the nature of these effects depends on whether *Wolbachia* and *Spiroplasma* are present singly or together in the host. Endosymbionts may mediate host thermoregulatory behavior and influence *Hsp* expression under temperature stress. These results suggest that endosymbionts affect thermal responses of mites and raise issues about how ongoing climate change might influence the distribution of endosymbionts as well as their hosts.

## Data Availability Statement

All sequence data have been submitted to the NCBI Sequence Read Archive, BioProject accession number PRJNA717652.

## Author Contributions

Y-XZ, AH, and X-YH designed the research. Y-XZ, Z-RS, Y-YZ, and X-YH performed the research. Y-XZ, Z-RS, AH, and X-YH wrote and edited the manuscript. All authors read and approved the manuscript.

## Conflict of Interest

The authors declare that the research was conducted in the absence of any commercial or financial relationships that could be construed as a potential conflict of interest.

## Footnotes

1 http://www.genome.jp/kegg/

2 http://www.geneontology.org/

## Supplementary Material

Supplementary Figure 1 Schematic representation of the study design. *w* + *s* + , *w* + , *s* +, and *w-s-* represent the spider mite strains infected with both *Wolbachia* and *Spiroplasma*, only *Wolbachia*, only *Spiroplasma*, and no endosymbionts, respectively. More than 282 mites from each strain were selected to test host Tp. The others were subjected to one of the four temperature treatments (20, 25, 30, and 35°C) for various time periods, depending on the experiment they were used for: (1) To assess survival rates at different temperatures, more than 116 female adults (2 days old) of each strain, grouped as six replicates of about 20 individuals, were exposed to each temperature treatment for a week with daily monitoring. (2) After 6 h of exposure to different temperatures, about 100 mites from each strain were collected for DNA extraction and transcriptome sequencing. (3) We collected mites exposed to different temperatures after 6, 12, 24, 72, and 120 h for testing densities of *Wolbachia* and *Spiroplasma*. (4) Each strain was reared for four generations under different temperature treatments. Twenty female adults were randomly selected from each combination of temperature and generation (F1, F2, F3, and F4) of these cultures, and used for PCR to detect the infection rates of *Wolbachia* and *Spiroplasma*. (5) In the crossing experiment, single females without *Wolbachia* (♀: *s* + or *w-s-*) in the teleiochrysalis stage were crossed with an adult virgin male (1 day old) infected with *Wolbachia* (♂: *w* + *s* + or *w* +) to produce four crosses. Mating and egg laying (up to 5 days) were carried out under one of the four temperature treatments, resulting in 16 total combinations, with 18–99 replicates each.

Supplementary Figure 2 **(A)** Schematic of the thermal gradient apparatus. **(B)** Plots showing the length of the 500 mm aluminum gradient and where temperature was recorded with K-type thermocouples. Red dots indicate spider mite entry point. **(C)** Linearity of temperature change for the different surfaces as measured with K-type thermocouples.

Supplementary Figure 3 Gene Ontology (GO) terms **(A)** and Kyoto Encyclopedia of Genes and Genomes (KEGG) terms **(B)** of genes identified from female *Tetranychus truncatus*.

Supplementary Figure 4 Confirmation of gene expression by quantitative real-time PCR. The relative expression level of each gene was determined by qPCR (red) and was compared with the expression of the transcriptomic data (blue).

Supplementary Table 1 Primers sequences. Sequences of DNA oligonucleotides used for assays described in this manuscript.

Supplementary Table 2 Information on *de novo* assembled genes of female spider mites and annotations to different databases.

Supplementary Table 3 Summarizes of endosymbiont infection pattern affects each of the host phenotypes.
