# Supplementary material for: Spider Mites Singly Infected With Either Wolbachia or Spiroplasma Have Reduced Thermal Tolerance
Source: Front Microbiol. 2021 Jul 7;12:706321. doi: 10.3389/fmicb.2021.706321 (PMC8292952; doi:10.3389/fmicb.2021.706321)
Supplement: Supplementary Figure 1 — Schematic representation of the study design. w + s + , w + , s +, and w-s- represent the spider mite strains infected with both Wolbachia and Spiroplasma, only Wolbachia, only Spiroplasma, and no endosymbionts, respectively. More than 282 mites from each strain were selected to test host Tp. The others were subjected to one of the four temperature treatments (20, 25, 30, and 35°C) for various time periods, depending on the experiment they were used for: (1) To assess survival rates at different temperatures, more than 116 female adults (2 days old) of each strain, grouped as six replicates of about 20 individuals, were exposed to each temperature treatment for a week with daily monitoring. (2) After 6 h of exposure to different temperatures, about 100 mites from each strain were collected for DNA extraction and transcriptome sequencing. (3) We collected mites exposed to different temperatures after 6, 12, 24, 72, and 120 h for testing densities of Wolbachia and Spiroplasma. (4) Each strain was reared for four generations under different temperature treatments. Twenty female adults were randomly selected from each combination of temperature and generation (F1, F2, F3, and F4) of these cultures, and used for PCR to detect the infection rates of Wolbachia and Spiroplasma. (5) In the crossing experiment, single females without Wolbachia (♀: s + or w-s-) in the teleiochrysalis stage were crossed with an adult virgin male (1 day old) infected with Wolbachia (♂: w + s + or w +) to produce four crosses. Mating and egg laying (up to 5 days) were carried out under one of the four temperature treatments, resulting in 16 total combinations, with 18–99 replicates each. [file Data_Sheet_1.docx]

***Supplementary Material***

**Spider Mites Singly Infected with Either *Wolbachia* or *Spiroplasma* Have Reduced Thermal Tolerance**

Yu-Xi Zhu^a,b^, Zhang-Rong Song^a^, Yi-Yin Zhang^a^, Ary A. Hoffmann^c^, Xiao-Yue Hong^a^*

^a.^ Department of Entomology, Nanjing Agricultural University, Nanjing, Jiangsu 210095, China.

^b.^ Institute of Applied Entomology, School of Horticulture and Plant Protection, Yangzhou University, Yangzhou, Jiangsu 225009, China.

^c.^ School of BioSciences, Bio21 Institute, The University of Melbourne, Victoria 3010, Australia

Running Head: **Endosymbionts and Spider Mite Thermal Tolerance**

*Correspondence to Xiao-Yue Hong, xyhong@njau.edu.cn, Tel: 025-84395339, Department of Entomology, College of Plant Protection, Nanjing Agricultural University, Nanjing, Jiangsu 210095, China

**This File Includes:**

**Supplementary Table 1- 3**

**Supplementary Figure 1 - 4**

**Supplementary TABLE 1∣** Primers sequences. Sequences of DNA oligonucleotides used for assays described in this manuscript.

|  | Species | Gene | Primer sequence (5'→3') | Annealing temp (℃) |
| --- | --- | --- | --- | --- |
| Diagnostic PCR | *Wolbachia* | wsp | wsp F:GTCCAATARSTGATGARGAAAC | 55 |
|  |  |  | wsp R:CYGCACCAAYAGYRCTRTAAA |  |
|  | *Spiroplasma* | 16S rRNA | SpitsJ04 F: GCCAGAAGTCAGTGTCCTAACCG | 56 |
|  |  |  | SpitsN55 R: ATTCCAAGGCATCCACCATACG |  |
| RT-qPCR | *Wolbachia* | wsp | wQF1: GAGCAGCGAATGTAAGCAATC | 60 |
|  |  |  | wQR1: AATAACGAGCACCAGCATAAAG |  |
|  | *Spiroplasma* | 16S rRNA | sQF1: TGTAGTTCTCAGGGATTGTTTTCTC | 60 |
|  |  |  | sQR1: CGCTTCCACCATCGCTCTT |  |
|  | Spider mite | CL1152.Contig1 | F: GTTGATCGGGGATGCTGCTA | 60 |
|  |  |  | R: TGAACTGTGGCATCGTCGAA |  |
|  |  | CL1469.Contig1 | F: CGCCTTGACCGGTTTTTACC | 60 |
|  |  |  | R: ATGCCAAGCGTCTAATCGGT |  |
|  |  | CL5629.Contig2 | F: GCAGCTGTTGAATTGGGTGC | 60 |
|  |  |  | R: CCGCTTCGATTTGCTTAGCC |  |
|  |  | Unigene5380 | F: GCACGATTCATTTGACCCCA | 60 |
|  |  |  | R: TGTCTCTTGTTCCACGACTCA |  |
|  |  | Unigene5382 | F: GCACGATTCATTTGACCCCA | 60 |
|  |  |  | R: TGTCTCTTGTTCCACGACTCA |  |
|  |  | CL635.Contig1 | F: TCTCCAACTCCTCCGATGCT | 60 |
|  |  |  | R: AGGGTGCGTTCATCTTGGTT |  |
|  |  | Unigene4246 | F: AAGAACGCCCATGTCTTCGT | 60 |
|  |  |  | R: CGGCCCATGATTGGAAGAGT |  |
|  |  | CL1127.Contig11 | F: ATTCCAAGGGTGCATAGCGA | 60 |
|  |  |  | R: CGCCGGTGATGATAGAGTCG |  |
|  |  | rps18 | rps18-F:ACGTGCTGGTGAACTTACCGAAGA | 60 |
|  |  |  | rps18-R:TGCCTATTCAAGAACCAAAGTGGG |  |

**Supplementary TABLE 2∣** Information on *de novo* assembled genes of female spider mites and annotations to different databases.

| **Main terms** | **Gene information and databases** | **Number** |
| --- | --- | --- |
| *De novo* assembled genes | Total clean bases (Gb) | 402.84 |
|  | Total clean reads (M) | 2685.47 |
|  | Clean reads Q30(%) | > 93.73 |
|  | Total number of unigenes | 57241 |
|  | Total length (bp) | 111142512 |
|  | Median length (bp) | 1941 |
|  | Unigenes larger than 3000 | 11169 |
|  | N50 | 2725 |
|  | N90 | 1067 |
|  | GC(%) | 35.25 |
| Annotated unigenes | NR | 44915 |
|  | NT | 17707 |
|  | GO | 28324 |
|  | KEGG | 37459 |
|  | Pfam | 37560 |
|  | KOG | 34817 |
| Annotation rate (%) | Annotated in all databases | 17.41 |
|  | Annotated in at least one database | 81.69 |

**Supplementary TABLE 3∣**Summarizes of endosymbiont infection pattern affects each of the host phenotypes.

| Spider mite strain | Spider mite phenotype | |
| --- | --- | --- |
|  | Thermal tolerance | Thermal preference |
| *w+s+* | High | Low temperature |
| *w+* | Low | Low temperature |
| *s+* | Low | High temperature |
| *w-s-* | High | High temperature |

*w+s+*, *w+*, *s+* and *w-s-* represent the spider mite strains infected with both *Wolbachia* and *Spiroplasma*, only *Wolbachia*, only *Spiroplasma* and no endosymbionts, respectively.


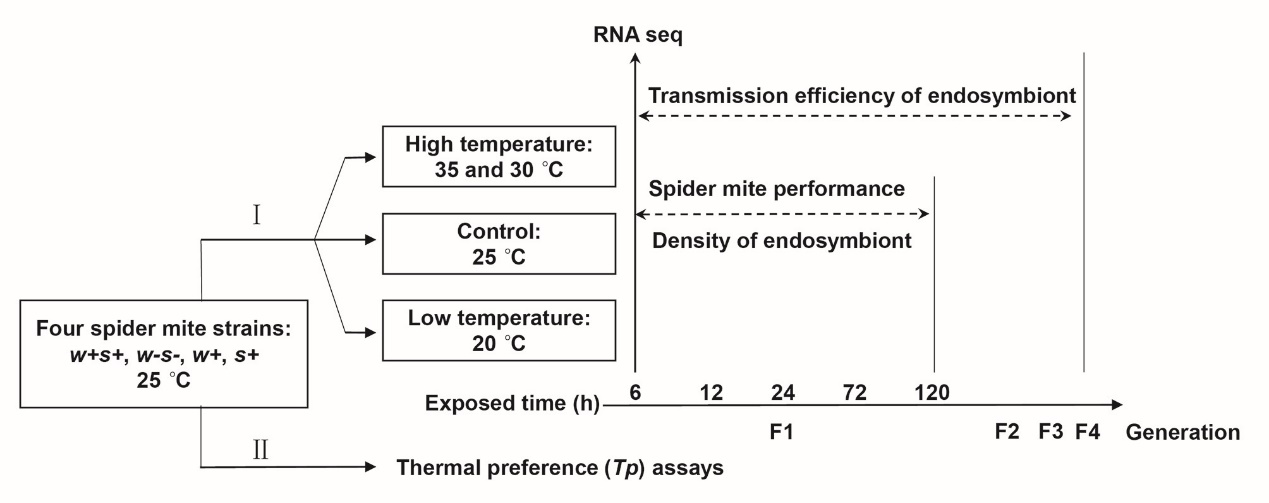


**Supplementary FIGURE 1∣** Schematic representation of the study design. *w+s+*, *w+*, *s+* and *w-s-* represent the spider mite strains infected with both *Wolbachia* and *Spiroplasma*, only *Wolbachia*, only *Spiroplasma* and no endosymbionts, respectively. More than 282 mites from each strain were selected to test host thermal preference. The others were subjected to one of four temperature treatments (20°C, 25°C, 30°C, and 35°C) for various time periods, depending on the experiment they were used for: 1) To assess survival rates at different temperatures, more than 116 female adults (2 days old) of each strain, grouped as 6 replicates of about 20 individuals, were exposed to each temperature treatment for a week with daily monitoring. 2) After 6 h of exposure to different temperatures, about 100 mites from each strain were collected for DNA extraction and transcriptome sequencing. 3) We collected mites exposed different temperatures after 6, 12, 24, 72 and 120 h for testing densities of *Wolbachia* and *Spiroplasma*. 4) Each strain was reared for four generations under different temperature treatments. Twenty female adults were randomly selected from each combination of temperature and generation (F1, F2, F3 and F4) of these cultures, and used for PCR to detect the infection rates of *Wolbachia* and *Spiroplasma*. 5) In the crossing experiment, single females without *Wolbachia* (♀: *s+* or *w-s-*) in the teleiochrysalis stage were crossed with an adult virgin male (1-day old) infected with *Wolbachia* (♂: *w+s+* or *w+*) to produce four crosses. Mating and egg laying (up to five days) were carried out under one of four temperature treatments, resulting in 16 total combinations, with 20-122 replicates each.


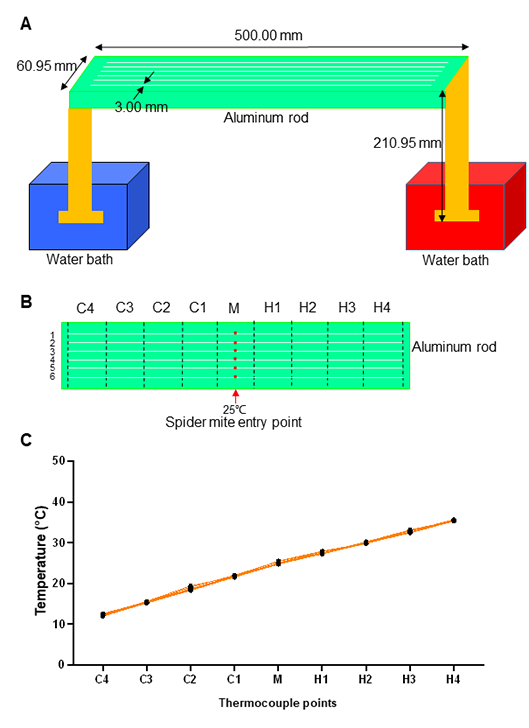


**Supplementary FIGURE 2∣** (**A**) Schematic of the thermal gradient apparatus. (**B**) Plots showing the length of the 500 mm aluminum gradient and where temperature was recorded with K-type thermocouples. Red dots indicate spider mite entry point. (**C**) Linearity of temperature change for the different surfaces as measured with K-type thermocouples.


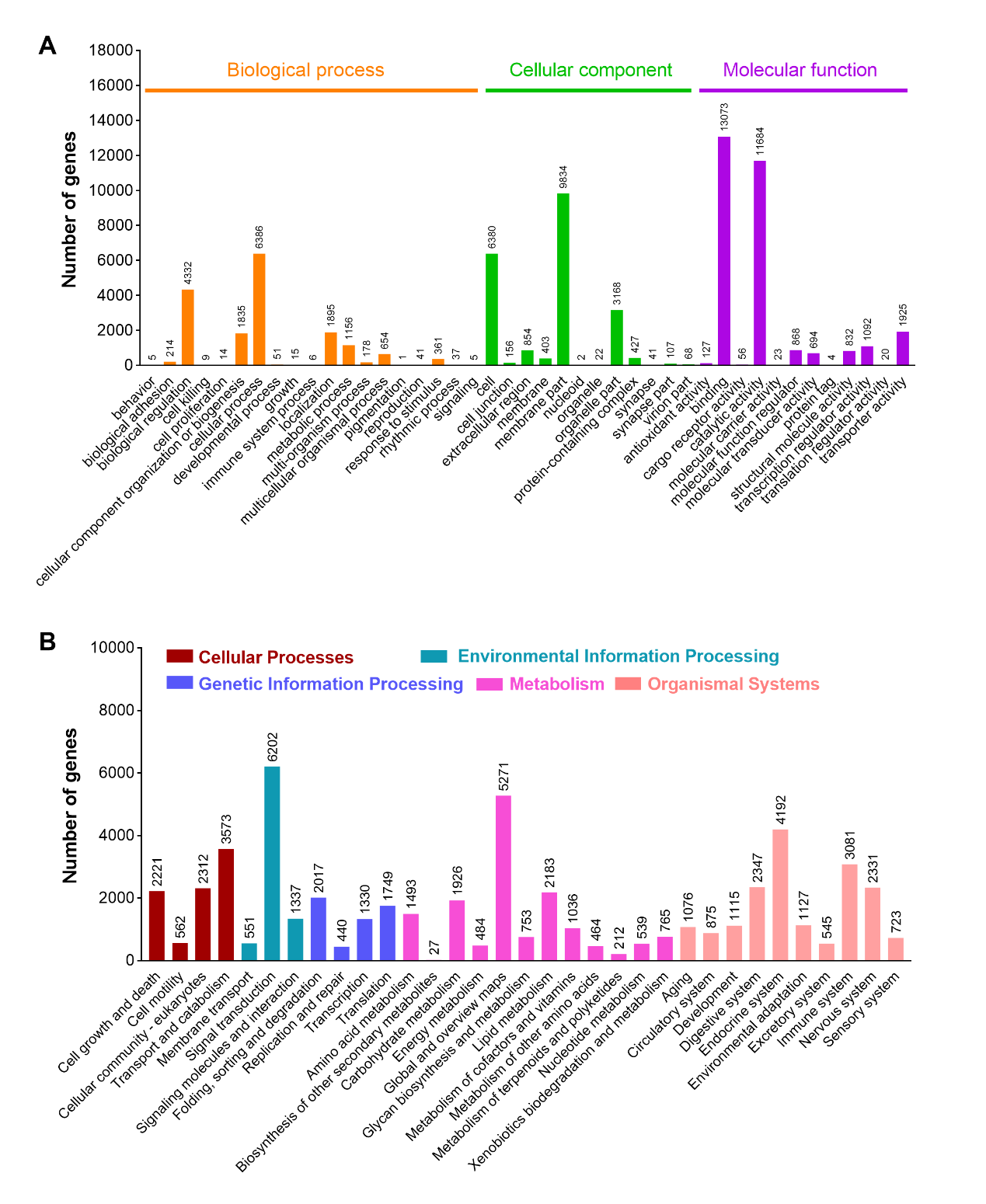


**Supplementary FIGURE 3∣** Gene Ontology (GO) terms (**A**) and Kyoto Encyclopedia of Genes and Genomes (KEGG) terms (**B**) of genes identified from female *Tetranychus truncatus*.


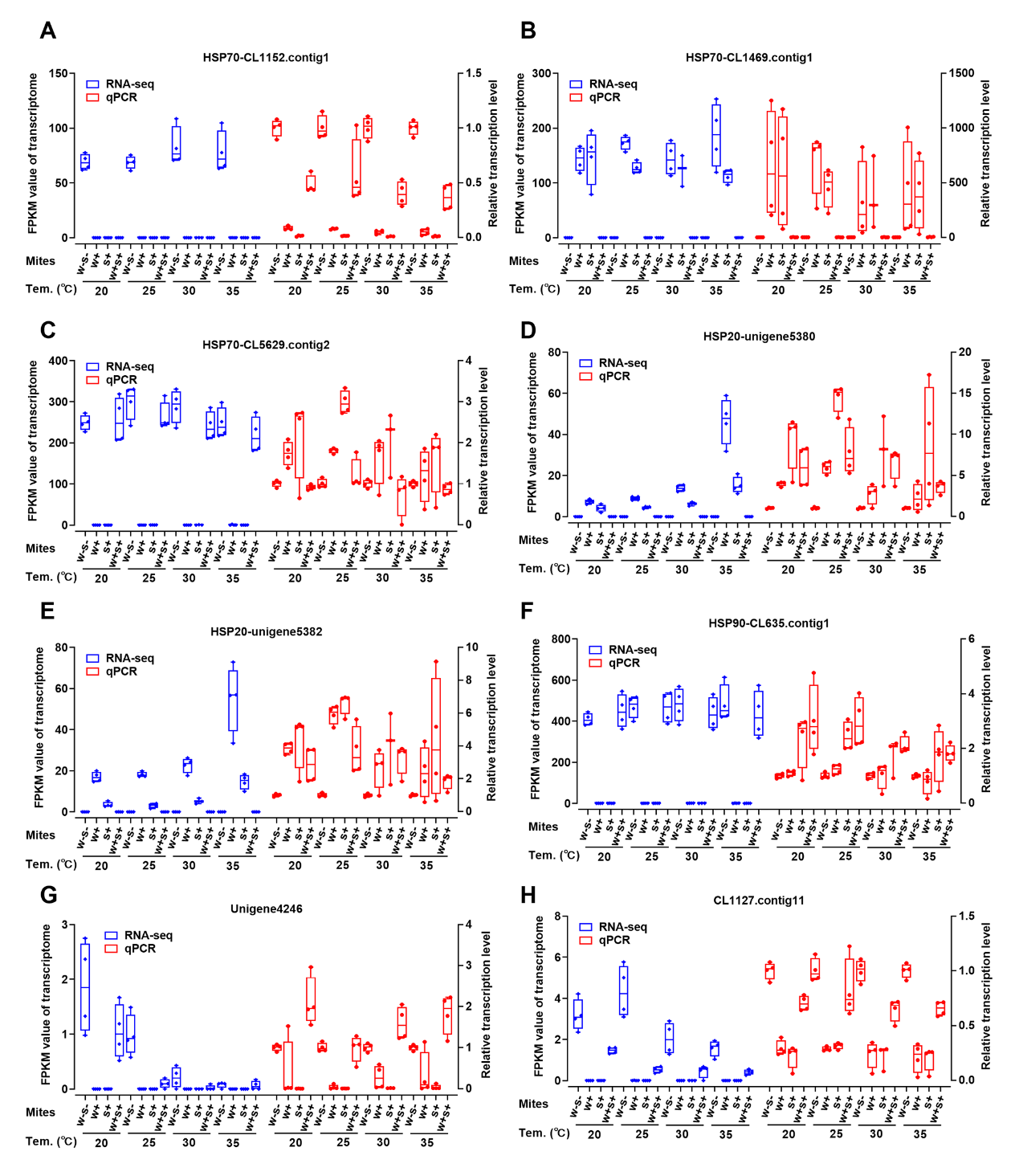


**Supplementary FIGURE 4∣** Confirmation of gene expression by quantitative real-time PCR. The relative expression level of each gene was determined by qPCR (red) and was compared with the expression of the transcriptomic data (blue).
